# Identification of high-impact chronic pain among cancer survivors: a cross-sectional study using the Graded Chronic Pain Scale framework

**DOI:** 10.1007/s00520-026-11190-z

**Published:** 2026-09-11

**Authors:** Julie Roed Jespersen, Henrik Bjarke Vaegter, Caroline Matilde Elnegaard, Lene Jarlbaek

**Affiliations:** 1 Pain Research Group, Pain Center, Department of Anaesthesiology and Intensive Care, Odense University Hospital, Odense, Denmark; 2 Department of Clinical Research, Faculty of Health Sciences, University of Southern Denmark, Odense, Denmark; 3 The Danish Knowledge Centre for Rehabilitation and Palliative Care (REHPA), Odense University Hospital, Nyborg, Denmark; 4 Department of Cardiology, Odense University Hospital, Odense, Denmark

**Keywords:** Cancer survivors, Chronic pain, Pain classification, High-impact pain, Rehabilitation, Interdisciplinary care

## Abstract

**Purpose:**

Chronic cancer-related pain is a prevalent and often under-recognized issue among individuals living with or after cancer. Clinically useful tools are needed to classify pain severity and impact and to identify those requiring targeted support. This study applied the Graded Chronic Pain Scale–Revised (GCPS-R) in a cohort of cancer survivors and examined differences in pain characteristics and psychosocial factors across GCPS-R subgroups. Secondly, the study compared pain characteristics in cancer survivors with high-impact chronic pain (HICP) to an age- and sex-matched sample of patients with non-cancer HICP.

**Methods:**

Patients attending a residential cancer rehabilitation course completed a screening questionnaire and a pain-specific module including the GCPS-R and validated measures of disability, psychological distress, pain catastrophizing, and self-rated health. Cancer survivors with HICP were matched 1:3 to patients with non-cancer HICP using registry data collected prior to their initial consultation at an interdisciplinary Pain Center.

**Results:**

Among 270 cancer survivors included in the analysis, 61% were classified as having HICP. Increasing GCPS-R impact levels were associated with greater pain intensity and disability, higher psychological distress and pain catastrophizing, and poorer self-rated health and quality of life (*p* < 0.05). Compared with matched non-cancer patients with HICP, cancer survivors reported lower pain intensity and catastrophizing but were at least as likely to meet criteria for widespread pain.

**Conclusion:**

HICP is present among cancer survivors seeking rehabilitation. The GCPS-R can differentiate clinically meaningful subgroups and may support systematic assessment of pain severity and impact to identify patients requiring targeted support.

**Supplementary Information:**

The online version contains supplementary material available at https://doi.org/10.1007/s00520-026-11190-z.

## Introduction

Chronic cancer-related pain can appear as a consequence of cancer or its treatment [1] and may be inadequately recognized and addressed [2, 3]. Studies have reported chronic pain in 28–47% of cancer survivors [4, 5], while US population-based data showed a prevalence of 31% among cancer survivors compared with 16% among individuals without a cancer history [6]. Pain may also co-occur with other persistent symptoms and late effects. In a recent Danish study of cancer survivors referred to a late effects clinic, pain was among the most frequently reported late effects alongside fatigue, cognitive impairment, sleep problems, and neuropathy, with most patients reporting multiple co-occurring late effects [7].

The consequences associated with chronic cancer-related pain are likely influenced by its severity and impact on daily life [8], emphasizing the importance of recognizing differences in pain impact. Routine assessment of pain characteristics is not common practice, highlighting the need for systematic classification frameworks to identify patients with high-impact chronic pain (HICP) who may require interdisciplinary multimodal pain therapy [3]. In a US survey of 4526 cancer survivors, 36% reported chronic pain, of whom 46% were categorized as having HICP [9].

HICP is defined as pain for at least 3 months with substantial impact on activities of daily living [8]. The Graded Chronic Pain Scale–Revised (GCPS-R) was developed to classify chronic non-cancer pain as mild, bothersome, or high-impact based on pain severity and impact [8, 10]. Given the limited use of the GCPS-R in cancer populations, its application may provide a clinically meaningful framework for characterizing variation in pain impact and associated burden among cancer survivors. An exploratory comparison with patients with non-cancer HICP may provide additional clinical context for interpreting the HICP subgroup.

### Aims

The primary aim of this study was to apply the GCPS-R in a population of cancer survivors to explore differences between patients classified as having mild, bothersome, or high-impact chronic cancer-related pain. The secondary, exploratory aim was to compare pain characteristics and psychosocial profiles between cancer survivors with HICP and a reference sample of patients with non-cancer HICP from an interdisciplinary Pain Center.

## Materials and methods

### Study design and participants

This cross-sectional study included cancer survivors reporting cancer-related pain who attended rehabilitation courses at the Danish Knowledge Center for Rehabilitation and Palliative Care (REHPA) from March 2022 to February 2025. In the present study, we use the term “cancer survivor” in accordance with previous cancer survivorship research to refer to individuals who have received a cancer diagnosis, regardless of where they are in the disease trajectory [11, 12].

REHPA offers structured rehabilitation courses comprising an initial 5-day residential stay, 7–10 weeks at home, and a final 2-day follow-up stay. The courses include group-based education and activities as well as individual guidance and address different cancer-related rehabilitation needs, including pain, fatigue, and other late effects. Participants were adults (≥ 18 years), self-reliant, and able to participate in the course, typically applying due to disease- or treatment-related rehabilitation needs. Applications required a physician referral including cancer diagnosis and rehabilitation needs. Disease stage, time since diagnosis, and cancer treatment were not systematically available in a format suitable for research purposes. Patients unable to complete questionnaires due to language barriers or cognitive impairment were excluded.

#### Study procedures and data collection

Data were collected from patients who reported pain in an initial screening questionnaire (Q), which was distributed 2–3 weeks prior to attending the rehabilitation course. The initial screening Q is part of REHPA’s routine data collection across rehabilitation courses and includes validated instruments covering various domains, including health-related quality of life, psychological distress, fatigue, and sleep quality, along with routinely collected items covering pain intensity and sociodemographic characteristics [13, 14].

Based on responses to the screening Q, patients meeting predefined pain criteria were identified and informed about the supplementary pain-specific questionnaire (“c-module”) during the initial 5-day residential stay at REHPA. Written informed consent for participation in the study was obtained during this stay. Consenting patients received a secure link to the c-module in the week following the residential stay. Eligibility required meeting at least one of the following four pain criteria:

1. Average pain intensity of ≥4 on a numeric rating scale over the past 2 days
2. Experiencing pain during the past week (“quite a bit” or “very much”)
3. Pain interfering with daily activities (“quite a bit” or “very much”)
4. Affirmed having pain on a daily, frequent, or occasional basis

as well as report that the pain was related to cancer and/or cancer treatment.

The c-module included validated instruments and items assessing pain characteristics, interference, location, and distribution. Several components were aligned with the national PainData Registry for chronic non-cancer pain [15], which contains questionnaire data collected before patients’ initial consultation at Danish interdisciplinary pain centers. This alignment enabled comparison of selected outcomes between cancer and non-cancer pain populations. All data were collected electronically and stored in accordance with data protection regulations.

### Measures

An overview of measures obtained from the initial screening questionnaire and the pain-specific c-module in the cancer cohort is provided in Table 1.

**Table 1:** Overview of measures obtained from the initial screening questionnaire and the pain-specific c-module

Measure domain	Data source	Instrument/item	Description and scoring
Sociodemographic characteristics	Initial screening Q	Standard item from routine REHPA data collection [14]	Age, sex (male/female), marital status (single/married/partnered), education (lower secondary or other/upper secondary/higher education)
Pain intensity (48 h)	Initial screening Q	Standard item from routine REHPA data collection [14]	Average pain intensity during the last 48 h, NRS 0 = no pain and 10 = worst possible pain
Pain during past week	Initial screening Q	Standard item from routine REHPA data collection [14]	1 = “Not at all,” 2 = “A little,” 3 = “Quite a bit,” and 4 = “Very much”
Pain interfering with daily activities	Initial screening Q	EORTC QLQ-C30 single item [16]	1 = “Not at all,” 2 = “A little,” 3 = “Quite a bit,” and 4 = “Very much”
Pain occurrence	Initial screening Q	Standard item from routine REHPA data collection [14]	Daily, frequent, occasional
Pain related to cancer diagnosis	Initial screening Q	Standard item from routine REHPA data collection [14]	Yes or no
Pain duration	Initial screening Q	Standard item from routine REHPA data collection [14]	< 3 months; 3–6 months; 6–12 months; > 1 year
Depression	Initial screening Q	PHQ-9 [17]	9 items; total score 0–27; higher scores indicate greater depressive symptoms
Anxiety	Initial screening Q	GAD-7 [18]	7 items; total score 0–21; higher scores indicate greater anxiety symptoms
Self-rated health	Initial screening Q	PROMIS Global Health single item [19]	1 (poor) to 5 (excellent)
Quality of life	Initial screening Q	EORTC QLQ-C30 single item [16]	1 (very poor) to 7 (excellent)
Chronic pain classification	c-module	GCPS-R [8]	Grade 0–3; mild, bothersome and HICP as described in Methods
Pain intensity (7 days)	c-module	GCPS-R [8]	Average pain intensity during the last 7 days, NRS 0 = no pain and 10 = worst possible pain
Widespread pain	c-module	Pain drawing [15]	71 body regions; widespread pain classified according to ACR 1990 criteria
Pain-related disability	c-module	PDI [20]	5 voluntary activity items; total score 0–50
Pain catastrophizing	c-module	PCS single item [21]	0 (never do that) to 10 (always do that)

Abbreviations: *Q* questionnaire, *REHPA* Danish Knowledge Centre for Rehabilitation and Palliative Care, *NRS* Numeric Rating Scale, *EORTC QLQ-C30* European Organisation for Research and Treatment of Cancer Quality of Life Numeric Rating Scale, *EORTC QLQ-C30* European Organisation for Research and Treatment of Cancer Quality of Life, *PROMIS* Patient-Reported Outcomes Measurement Information System, *GCPS-R* Graded Chronic Pain Scale–Revised, *HICP* high-impact chronic pain, *ACR* American College of Rheumatology, *PDI* Pain Disability Index, *PCS* Pain Catastrophizing Scale

### Initial screening questionnaire

The initial screening questionnaire included sociodemographic characteristics and some measures of pain, psychological distress, self-rated health, and quality of life. Pain-related items assessing pain intensity, pain interference, and pain frequency were used to determine eligibility for the supplementary pain-specific “c-module.”

*Sociodemographic variables* included age, sex, marital status, education level, smoking status, alcohol consumption, and physical activity level. Pain duration was assessed categorically (< 3 months, 3–6 months, 6–12 months, or > 1 year).

*Symptoms of depression* were assessed using the *Patient Health Questionnaire-9* (PHQ-9). The scale consists of nine items related to core symptoms of depression, each rated on a 4-point Likert scale from 0 = “not at all” to 3 = “nearly every day” yielding a total score ranging from 0 to 27. Higher scores indicate greater depression severity [22]. The PHQ-9 is a validated measure of depressive symptoms [17] and is widely used in chronic pain populations [23].

*Symptoms of anxiety* were assessed using the *Generalized Anxiety Disorder-7* (GAD-7) questionnaire. The GAD-7 comprises seven items rated on a 4-point Likert scale from 0 = “not at all” to 3 = “nearly every day” with a total score ranging from 0 to 21. Higher scores reflect more severe anxiety symptoms [18]. The GAD-7 is a validated measure of anxiety symptoms and is commonly applied in studies involving patients with chronic pain [23].

*Self-rated health* was assessed using the PROMIS Global Health item, which asks patients to rate their general health on a 5-point Likert scale ranging from 1 (poor) to 5 (excellent). This single item has shown acceptable validity as a global measure of perceived health status in clinical and population-based samples [19].

*Quality of life* was assessed using a single item from the EORTC QLQ-C30, asking patients: “How would you rate your overall quality of life during the past week?” Responses were scored on a 7-point Likert scale ranging from 1 (very poor) to 7 (excellent), with higher scores indicating better quality of life [16].

#### “The c-module”

The pain-specific “c-module” included the GCPS-R, pain drawing, the Pain Disability Index (PDI), and a single item from the Pain Catastrophizing Scale (PCS), assessing pain characteristics and impact, pain distribution, pain-related disability, and pain-related cognition.

*GCPS-R*: The GCPS-R is a brief, self-administered six-item questionnaire that classifies chronic pain hierarchically as Grade 0 (chronic pain absent), Grade 1 (mild chronic pain), Grade 2 (bothersome chronic pain), or Grade 3 (HICP) [8]. Chronic pain is defined as pain on most days or every day during the past 3 months. Among participants with chronic pain, HICP is defined by pain limiting life activities or work on most days or every day; the remaining participants are classified as bothersome or mild chronic pain based on the three-item Pain, Enjoyment and General Activities (PEG) scale, using a cut-off score of ≥ 12 [8].

*Widespread pain*: Based on patients’ pain drawing (Supplementary Fig. 1) on an anatomically divided body with 71 regions [15], widespread pain was coded according to the American College of Rheumatology 1990 criteria, defined as pain present on both the left and right sides of the body, above and below the waist, and in the axial skeleton (neck, back, or chest) [24].

*Pain-related disability* was assessed using the *Pain Disability Index* (PDI), measuring pain interference with voluntary daily activities [25]. Five items are scored from 0 (“no disability”) to 10 (“worst disability”), yielding a total score of 0–50. In line with previous psychometric analyses, the five voluntary activity items were included, while the two obligatory activity items (self-care and life-support activities) were excluded [20].

*Catastrophic thinking* related to pain was assessed using a single item from the *Pain Catastrophizing Scale* (PCS): “When I am in pain, it’s terrible and I think it’s never going to get any better.” Responses were scored on a scale from 0 = “never do that” to 10 = “always do that.” The item was selected based on previous studies demonstrating its predictive validity for pain-related outcomes [21].

### Comparison sample from interdisciplinary Pain Center

For the secondary, exploratory aim, an external reference sample of patients with non-cancer HICP was extracted from the PainData Registry at the interdisciplinary Pain Center, Odense University Hospital. A stratified random sampling procedure was applied to create a dataset with a 1:3 ratio of cancer to non-cancer patients, with matching performed without replacement. Based on age and sex, three non-cancer patients were randomly selected for each cancer patient within each of the following 10-year age strata: 18–29, 30–39, 40–49, 50–59, 60–69, 70–79, and 80–89. Between-group analyses included variables measured comparably in both cohorts: pain drawing, average pain intensity during the past week (NRS 0–10), depression (PHQ-9), anxiety (GAD-7), pain catastrophizing (single PCS item), and self-rated health (PROMIS Global Health single item). Pain duration was available in both cohorts but assessed differently: categorically in the cancer cohort and as time since pain onset in the PainData Registry. It was therefore described separately and excluded from between-group analysis.

### Statistical analysis

Descriptive statistics were used to summarize patient characteristics and questionnaire scores. For the primary aim, differences between the three GCPS-R categories were assessed using one-way analysis of variance (ANOVA) for continuous variables, followed by Bonferroni-adjusted pairwise comparisons when the overall test was significant (*p* < 0.05). Only patients with complete GCPS-R data were included. Screening-Q responses were compared between c-module responders and non-responders to explore potential selection bias.

For the secondary exploratory aim, unpaired *t*-tests and chi-square tests were used to compare patients with cancer-related HICP and non-cancer HICP. Effect sizes were calculated using Cohen’s *d*, with values around 0.2, 0.5, and ≥ 0.8 indicating small, moderate, and large effects, respectively [26].

All statistical analyses were conducted using STATA version 15 [27].

## Results

**Fig. 1:**
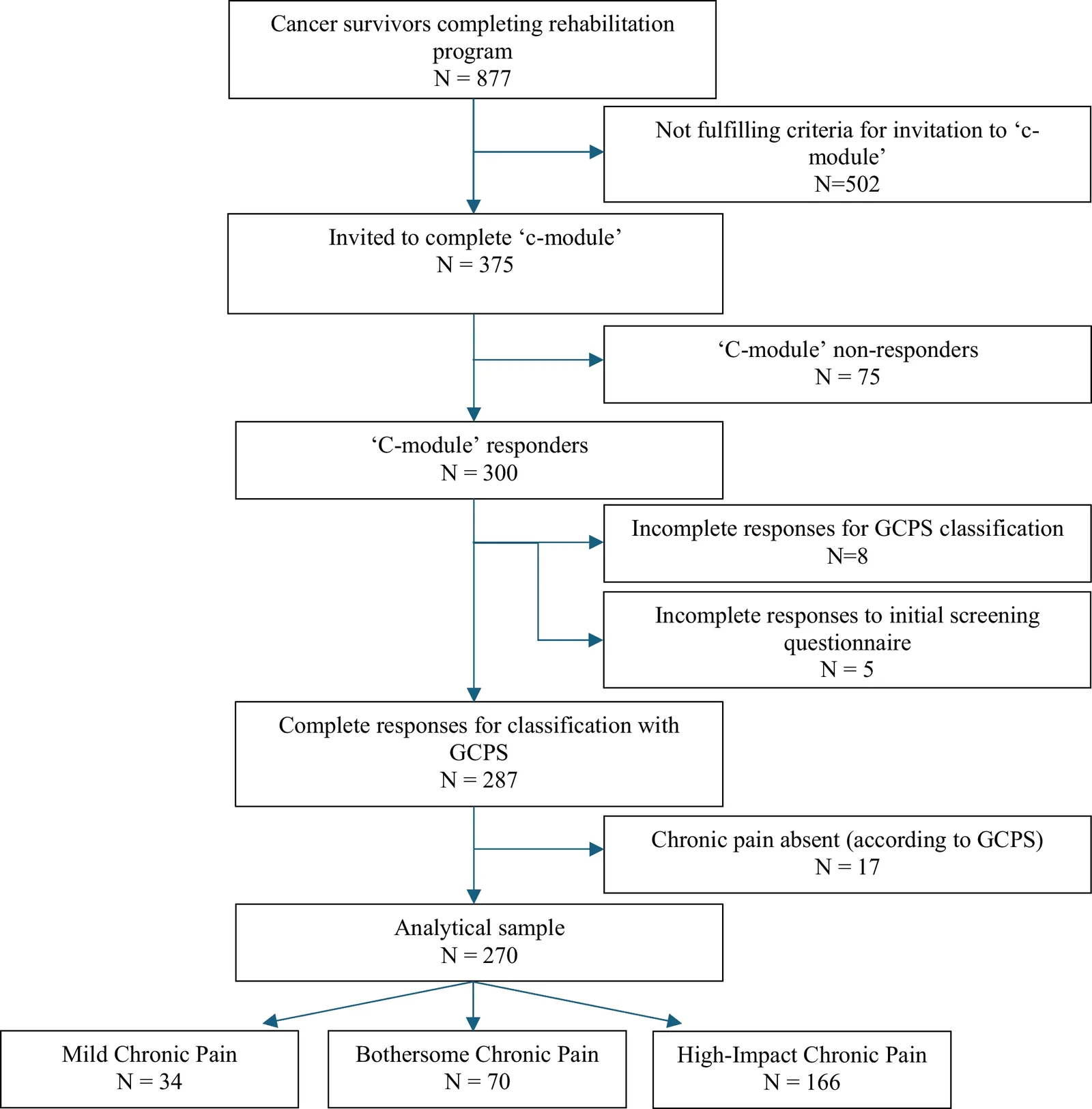
Flowchart of participant inclusion and pain classification in the REHPA cohort

Figure 1 illustrates the participant flow of the cancer survivor cohort.

Of the 877 cancer survivors who participated in the cancer rehabilitation course at REHPA, 375 met the pain criteria for receiving the “c-module.” Of these, 300 responded. Non-responders were slightly younger and reported marginally lower pain intensity and pain interference, as well as lower levels of physical activity, but did not differ substantially from responders on other clinical or demographic variables (see supplementary Table 1).

Of the 300 respondents, 5 had not completed the initial questionnaire, 8 had insufficient GCPS-R data to allow classification, and 17 were classified as having chronic pain absent according to the GCPS-R. The final study sample therefore comprised 270 patients with chronic pain, classified as mild (13%), bothersome (26%), or high-impact (61%) chronic pain (Table 2). The underlying cancer diagnoses among the 270 patients were as follows: 58% with breast cancer, 9% with gastrointestinal cancer, 9% with hematological cancer, mainly multiple myeloma, and the remaining 24% had various other cancer diagnoses.

**Table 2:** Demographics and clinical characteristics of cancer survivors according to GCPS-R chronic pain grade

		Total n= 270		Mild n=34		Bothersome n = 70		High Impact n=166		Analysis of variance (ANOVA)			
										ANOVA, p	Mild vs. Bothersome, p	Mild vs. High Impact, p	Bothersome vs. High impact, p
**Demographics**	**Age (years) **												
	Mean (95% CI)	55.2	(53.9; 56.6)	56.1	(52.5; 59.7)	54.7	(52.1; 57.4)	55.3	(53.6; 57.0)	0.8			
	**Sex**												
	% Female	89		91.2		85.7		89.8					
**Pain**	**Pain duration at initial screening **												
	< 3 months n, %	23	8.5%	6	17.7%	6	8.6%	11	6.6%	0.03	1	0.09	0.04
	3–6 months n, %	34	12.6%	<5	<10%	13	18.6%	18	10.8%				
	6–12 months n, %	64	23.7%	11	32.4%	20	28.6%	33	19.9%				
	>1 year n, %	148	54.8%	13	38.2%	31	44.3%	104	62.7%				
	**Pain intensity (past week)**												
	Mean (95% CI)	5.7	(5.5; 5.9)	3.7	(3.3; 4.1)	5.8	(5.4; 6.1)	6.1	(5.9; 6.4)	0.00	0.00	0.00	0.32
	Mild (1–3) n, %	34	12.6%	18	52.9%	<5	<5%	13	7.8%	0.00	0.00	0.00	0.4
	Moderate (4–6) n, %	137	50.7%	15	44.1%	46	65.7%	76	45.8%				
	Severe (>=7) n, %	99	36.7%	<5	<5%	21	30.0%	77	45.8%				
	**Pain distribution, (0–71 regions)**												
	Mean (95% CI)	19.1	(17.7; 20.6)	15.7	(11.6; 19.9)	17.6	(15.3; 19.9)	20.5	(18.6; 22.4)	0.04	1	1	0.57
	**Widespread pain (WP 1990)**												
	n, %	164	60.7%	16	47.1%	38	54.3%	110	66.3%	0.05	1	0.11	0.25
**Disability**	**Daily activities limited by pain (past week)**												
	Not at all n, %	32	11.9%	10	29.4%	12	17.1%	10	6%	0.00	0.28	0.00	0.00
	A little n, %	134	49.6%	21	61.8%	42	60%	71	42.8%				
	Quite a bit n, %	82	30.4%	<5	<10%	14	20%	66	39.8%				
	Very much n, %	22	8.2%	<5	<5%	<5	<5%	19	11.5%				
	**Pain disability index (PDI 5 items: 0–50)**												
	Mean (95% CI)	25.6	(24.3; 26.8)	13.8	(11.0; 16.6)	22.0	(20; 24.1)	29.5	(28.1; 30.8)	0.00	0.00	0.00	0.00
**Health & QoL**	**Self rated health (PROMIS: 1–5) higher is better**												
	Mean (95% CI)	2.3	(2.3; 2.4)	2.6	(2.3; 2.8)	2.5	(2.3; 2.7)	2.2	(2.1; 2.3)	0.00	1	0.03	0.00
	**Self rated quality of life past week (0–7) higher is better **												
	Mean (95% CI)	4.1	(4; 4.3)	4.6	(4.2; 5.1)	4.2	(3.9; 4.5)	4	(3.9; 4.2)	0.01	0.15	0.01	0.89
**Psychological**	**Depression (PHQ9: 0–27) higher is worse**												
	Mean (95% CI)	8.9	(8.4; 9.4)	6.8	(5.5; 8.2)	8.7	(7.7; 9.7)	9.4	(8.7; 10.1)	0.01	0.11	0.01	0.79
	**Anxiety (GAD7: 0–21) higher is worse **												
	Mean (95% CI)	5.6	(5.1; 6.1)	4.5	(3.3; 5.7)	5.5	(4.5; 6.6)	5.8	(5.1; 6.5)	0.27			
	**Pain catastrophizing (PCS Question 3, 0–10) higher is worse**												
	Mean (95% CI)	5.1	(4.8; 5.4)	3.4	(2.7; 4.0)	5	(4.5; 5.6)	5.5	(5.2; 5.9)	0.00	0.00	0.00	0.41

The three GCPS-R groups were similar in age, gender, marital status, educational background, and health behavior (Supplementary Table 2). Overall, 54.8% reported pain for more than 1 year, with a higher proportion in the HICP group (62.7%) than in the bothersome (44.3%) and mild chronic pain groups (38.2%) (*p* = 0.03).

### Pain characteristics, psychological distress, health, and QoL

Patients with HICP reported significantly higher pain intensity and disability, higher depression scores, more widespread pain, and lower self-rated health and quality of life compared with the bothersome and mild groups (all *p* < 0.05) (Table 2). Mean pain intensity corresponded to moderate-to-severe levels in the HICP group, moderate in the bothersome group, and low in the mild group. Widespread pain was reported by approximately two-thirds of patients with HICP. Pain catastrophizing and disability scores were also significantly higher in the HICP and bothersome groups compared with the mild chronic pain group.

### Comparison of cancer survivors with HICP and matched patients with HICP from the Pain Center

Of the 270 cancer survivors in the REHPA cohort, 166 were classified as having HICP and were included in the 1:3 matched comparison with 498 patients with HICP from the interdisciplinary Pain Center. Among the Pain Center patients with available data on pain duration, 94.2% had experienced pain for more than 1 year. The median pain duration was 7.6 years (IQR 2.7–16.0), and the mean was 11.1 years (95% CI 10.1; 12.1).

Compared with HICP cancer survivors, patients from the interdisciplinary Pain Center reported higher scores on pain intensity (*d* = 1.03, *p* < 0.01), pain-related disability (*d* = 0.80, *p* < 0.01), depressive symptoms (*d* = 0.27, *p* < 0.01), and pain catastrophizing (*d* = 0.49, *p* < 0.01), larger pain distribution (*d* = 0.24, *p* < 0.05), and lower self-rated health (*d* = 0.64, *p* < 0.01) (Table 3). No significant difference was observed in anxiety symptoms (GAD-7; *d* = 0.05, *p* = 0.61).

**Table 3:** Comparison of non-cancer patients with high-impact chronic pain and cancer survivors with high-impact chronic pain

		Non-cancer HICP *n* = 498		Cancer HICP *n* = 166		*t*-test, *p*	Effect size, *d*
**Demographics**	**Age (years)**						
	Mean (95% CI)	54.6	(53.6; 55.6)	55.3	(53.6; 57.0)		
	**Sex**						
	% female	89.8		89.76			
**Pain**	**Pain intensity (during past week)**						
	Mean (95% CI)	7.7	(7.6; 7.8)	6.1	(5.9; 6.4)	0.00	1.03
	**Pain distribution (0–71 regions)**						
	Mean (95% CI)	24.5	(23.0; 26.1)	20.5	(18.6; 22.4)	0.01	0.24
	**Widespread pain (WP 1990)**						
	*n*, %	289	58.0%	110	66.3%	0.06	RR = 1.14
**Disability**	**Pain disability index (PDI 5 items: 0–50) higher is worse**						
	Mean (95% CI)	36.4	(35.7; 37.2)	29.5	(28.1; 30.8)	0.00	0.80
**Health**	**Self-rated health (PROMIS: 1–5) higher is better**						
	Mean (95% CI)	1.8	(1.7; 1.8)	2.2	(2.1; 2.3)	0.00	0.64
**Psychological**	**Depression (PHQ9: 0–27) higher is worse**						
	Mean (95% CI)	10.8	(10.3; 11.3)	9.4	(8.7; 10.1)	0.00	0.27
	**Anxiety (GAD7: 0–21) higher is worse**						
	Mean (95% CI)	6.0	(5.6; 6.5)	5.8	(5.1; 6.5)	0.61	0.05
	**Pain catastrophizing (PCS Question 3, 0–10) higher is worse**						
	Mean (95% CI)	6.7	(6.5; 7.0)	5.5	(5.2; 5.9)	0.00	0.49

## Discussion

In this cancer rehabilitation cohort, most participants reporting pain met the GCPS-R criteria for chronic pain, with HICP being the most common classification and associated with greater functional and psychosocial burden. Compared with matched patients with non-cancer HICP, cancer survivors with HICP generally reported lower symptom burden, although widespread pain was more prevalent.

### Characteristics of cancer survivors with high-impact pain

The high prevalence of chronic pain and HICP aligns with prior research on long-term pain among cancer survivors, both in rehabilitation settings [13] and in population-based samples [6]. Patients in the high-impact group reported higher pain intensity, pain distribution, disability, psychological distress, and pain-related cognitions, alongside lower self-rated health and quality of life compared with mild and bothersome chronic cancer pain groups. These findings should be considered within the broader symptom burden experienced by cancer survivors, where pain often co-occurs with other persistent symptoms and late effects [7, 28]. Glare et al. reported moderate levels of pain intensity and interference, psychological distress, and pain catastrophizing among cancer survivors referred to a multidisciplinary pain clinic [29]. However, direct comparison of symptom levels across studies remains difficult due to differences in patient selection, pain classification, and the instruments used to assess psychological and functional outcomes. Studies directly comparing cancer-related and non-cancer chronic pain populations using the same definition of HICP and comparable symptom measures appear to be scarce.

The observed differences across GCPS-R groups suggest that the classification can distinguish levels of pain impact within this cancer rehabilitation population and may help identify survivors with greater functional and psychosocial burden who could benefit from more intensive or targeted support.

### Comparing high-impact chronic pain across diagnostic contexts

To contextualize HICP in cancer survivorship, we exploratively compared cancer survivors with HICP with matched patients with non-cancer HICP referred to an interdisciplinary Pain Center. Despite meeting the same impact criteria, the two groups differed on several indicators. The non-cancer group reported significantly higher pain intensity and greater pain catastrophizing, although levels were elevated in both groups.

Although the non-cancer group reported a higher number of pain areas, cancer survivors were more likely to fulfill the criteria for widespread pain. This pattern may reflect differences in the spatial distribution of pain rather than the number of pain sites. One possible hypothesis is that chemotherapy-induced peripheral neuropathy—often affecting both upper and lower extremities—is frequent among cancer survivors, and it may have contributed to this pattern [7,. Other treatment-related contributors, such as aromatase inhibitors and surgical late effects, have also been associated with persistent, diffuse pain [4, 5, 7].

These differences may reflect underlying pain mechanisms and diagnostic classifications. According to the ICD-11, chronic cancer-related pain is categorized as chronic secondary pain, attributable to a known disease or treatment, whereas the non-cancer group may comprise patients with chronic primary pain, where pain is the main problem and often lacks a clear biomedical cause [15, 31]. These typological distinctions may shape symptom interpretation and coping responses. Hellmann et al. [32] similarly emphasized that although high-impact pain is consistently associated with psychological burden and adverse lifestyle factors, its clinical presentation and underlying drivers differ across populations.

### Implications for rehabilitation and care planning

The findings underline the need for systematic assessment of pain impact, not just its presence, in cancer rehabilitation. HICP was common and associated with greater psychological and functional burden. Yet, chronic cancer-related pain remains poorly assessed and treated in many clinical settings, despite having its own diagnostic code in the ICD-11.

The use of tools like the GCPS-R may help to identify those survivors in the most need of intensified support. Acknowledging the heterogeneity within cancer-related HICP, there is a need for more responsive, interdisciplinary care strategies that address the complexity of chronic pain experiences in cancer survivors. Cancer survivors with HICP may require guidance and tailored interventions that extend beyond standard biomedical approaches.

The need for interdisciplinary care is also supported by Hofbauer et al. [3], who emphasize the importance of improving access to interdisciplinary multimodal pain therapy for patients living with chronic cancer-related pain. The differences observed between the cancer-related and non-cancer pain profiles in this study further suggest that HICP is a heterogeneous construct, influenced by underlying pain mechanisms and contextual factors. These findings raise important questions about whether access to cancer-specific pain expertise should be strengthened—an issue that calls for further clinical research.

### Strengths and limitations

This study was conducted in a selected population of cancer survivors, who applied for rehabilitation in a single center and who reported pain. This selection limits the generalizability of the findings to the broader population of cancer survivors; however, the sample represents a clinically relevant segment of patients living with or after cancer who experience rehabilitation needs related to cancer or its treatment. The high proportion of women in the study population further limits generalizability, although an overrepresentation of women has also been reported in other cancer rehabilitation populations [7, 33]. Strengths of the study include its prospective design, the relatively large sample size, and low levels of missing data.

The use of a well-characterized, age- and sex-matched comparison group of patients with non-cancer HICP was also a strength. A comparison between the “c-module” responders and non-responders, based on their responses to the screening Q did not reveal any striking differences in demographic or clinical characteristics, which reduces the risk of systematic non-response bias. In this context, the GCPS-R provided a structure for identifying variation in impact levels that may otherwise be overlooked.

However, some limitations should be considered. First, the study population consisted of patients who actively applied for rehabilitation due to a variety of cancer-related problems, including, but not limited to, pain. This selection may result in selection bias toward more resourceful or motivated individuals. Furthermore, detailed cancer-related characteristics, including time since diagnosis, disease stage, and previous and current antineoplastic treatment, were not systematically available. The cohort included participants living with incurable cancer; however, disease stage was not systematically recorded and could therefore not be included in the analyses. Consequently, we were unable to examine whether these factors were associated with pain characteristics or chronic pain severity.

Second, pain problems were categorized as cancer-related based on patient-reported data and were not verified by clinical assessment. Although self-reported information on comorbidities was collected, these data were considered insufficiently reliable to determine whether other conditions may have contributed to chronic pain. Thus, some patients may have had pre-existing or concurrent pain conditions unrelated to their cancer or treatment. Information on ongoing pharmacological and non-pharmacological pain management was not systematically available and could therefore not be considered when interpreting differences between the populations.

Finally, pain duration of ≥ 3 months was not an eligibility criterion for the pain-specific module. Consequently, some participants with a self-reported pain duration of < 3 months fulfilled the GCPS-R frequency criterion of pain on most days or every day during the previous 3 months and were classified with chronic pain.

#### Conclusion

In this rehabilitation cohort, 61% of included cancer survivors reporting pain were classified as having HICP using the GCPS-R. The GCPS-R classification identified distinct psychological and functional profiles across chronic pain severity groups. Increasing GCPS-R impact levels were associated with greater pain intensity, disability, psychological distress, and pain catastrophizing, as well as worse self-rated health and quality of life.

Compared with patients with non-cancer HICP, cancer survivors with HICP reported lower pain intensity and catastrophizing but were more likely to meet criteria for widespread pain. These findings support the clinical relevance of systematically assessing pain impact in cancer survivorship and using structured classification to identify patients in need of targeted support.

## Supplementary Information

Below is the link to the electronic supplementary material.

ESM 1 (DOCX 645 KB)

## Data Availability

The data underlying this study are not publicly available due to their individual-level nature, the associated patient confidentiality and data protection regulations. The data are therefore not available upon request.

## References

[1] Bennett MI et al (2019) Standards for the management of cancer-related pain across Europe-a position paper from the EFIC Task Force on Cancer Pain. Eur J Pain 23(4):660–668 10.1002/ejp.1346 PMC7027571 30480345

[2] Check DK et al (2023) Clinician perspectives on managing chronic pain after curative-intent cancer treatment. JCO Oncol Pract 19(4):e484–e491 10.1200/OP.22.00410 PMC10530392 36595729

[3] Hofbauer H et al (2025) Treatment satisfaction and treatment wishes of patients with chronic cancer-related pain. Oncol Res Treat 48(7–8):437–447 10.1159/000545363 40122040

[4] Pérez C et al (2024) Pain in long-term cancer survivors: prevalence and impact in a cohort composed mostly of breast cancer survivors. Cancers. 10.3390/cancers16081581 PMC11049399 38672663

[5] Bouhassira D, Luporsi E, Krakowski I (2017) Prevalence and incidence of chronic pain with or without neuropathic characteristics in patients with cancer. Pain 158(6):1118–1125 10.1097/j.pain.0000000000000895 28267066

[6] Sanford NN et al (2019) Prevalence of chronic pain among cancer survivors in the United States, 2010–2017. Cancer 125(23):4310–4318 10.1002/cncr.32450 31436323

[7] Tolstrup LK et al (2024) Health-related quality of life in Danish cancer survivors referred to a late effects clinic: a prospective cohort study. Acta Oncol 63:426–432 10.2340/1651-226X.2024.39937 PMC11332500 38881340

[8] Von Korff M et al (2020) Graded chronic pain scale revised: mild, bothersome, and high-impact chronic pain. Pain 161(3):651–661 10.1097/j.pain.0000000000001758 PMC7097879 31764390

[9] Jiang C et al (2019) Prevalence of chronic pain and high-impact chronic pain in cancer survivors in the United States. JAMA Oncol 5(8):1224–1226 10.1001/jamaoncol.2019.1439 PMC6587142 31219506

[10] Hellmann SS et al (2025) Graded chronic noncancer pain distribution using the Graded Chronic Pain Scale-Revised framework: a cross-sectional study. PAIN Rep. 10.1097/PR9.0000000000001277 PMC12119051 40444026

[11] Paice JA et al (2016) Management of chronic pain in survivors of adult cancers: American Society of Clinical Oncology Clinical Practice Guideline. J Clin Oncol 34(27):3325–3345 10.1200/JCO.2016.68.5206 27458286

[12] Wagle NS et al (2025) Cancer treatment and survivorship statistics, 2025. CA Cancer J Clin 75(4):308–340 10.3322/caac.70011 PMC12223361 40445120

[13] Jespersen E et al (2021) Everyday living with pain – reported by patients with multiple myeloma. Scand J Pain 21(1):127–134 10.1515/sjpain-2020-0087 33108340

[14] Elnegaard CM et al (2023) REHPA Forskningsdatabase, Metode, variabelbeskrivelse og forskningsmuligheder. REHPA, notat nr :26. REHPA, Videncenter for Rehabilitering og Palliation, Nyborg. https://www.rehpa.dk/wp-content/uploads/2023/12/REHPA-notat-26-REHPA-Forskningsdatabase.-Metode-variabelbeskrivelse-og-forskningsmuligheder.pdf

[15] Vaegter HB et al (2021) Socio-demographics, pain characteristics, quality of life and treatment values before and after specialized interdisciplinary pain treatment: results from the Danish Clinical Pain Registry (PainData). J Pain Res 14:1215–1230 10.2147/JPR.S306504 PMC8106464 33976571

[16] Kaasa S et al (1995) The EORTC core quality of life questionnaire (QLQ-C30): validity and reliability when analysed with patients treated with palliative radiotherapy. Eur J Cancer 31a(13–14):2260–2263 10.1016/0959-8049(95)00296-0 8652253

[17] Löwe B et al (2004) Measuring depression outcome with a brief self-report instrument: sensitivity to change of the Patient Health Questionnaire (PHQ-9). J Affect Disord 81(1):61–66 10.1016/S0165-0327(03)00198-8 15183601

[18] Spitzer RL et al (2006) A brief measure for assessing generalized anxiety disorder: the GAD-7. Arch Intern Med 166(10):1092–1097 10.1001/archinte.166.10.1092 16717171

[19] Cella D et al (2010) The Patient-Reported Outcomes Measurement Information System (PROMIS) developed and tested its first wave of adult self-reported health outcome item banks: 2005–2008. J Clin Epidemiol 63(11):1179–1194 10.1016/j.jclinepi.2010.04.011 PMC2965562 20685078

[20] Soer R et al (2013) Extensive validation of the pain disability index in 3 groups of patients with musculoskeletal pain. Spine (Phila Pa 1976) 38(9):E562–E568 10.1097/BRS.0b013e31828af21f 23388675

[21] Rosenstiel AK, Keefe FJ (1983) The use of coping strategies in chronic low back pain patients: relationship to patient characteristics and current adjustment. Pain 17(1):33–44 10.1016/0304-3959(83)90125-2 6226916

[22] Kroenke K, Spitzer RL, Williams JB (2001) The PHQ-9: validity of a brief depression severity measure. J Gen Intern Med 16(9):606–613 10.1046/j.1525-1497.2001.016009606.x PMC1495268 11556941

[23] Bair MJ et al (2008) Association of depression and anxiety alone and in combination with chronic musculoskeletal pain in primary care patients. Psychosom Med 70(8):890–897 10.1097/PSY.0b013e318185c510 PMC2902727 18799425

[24] Wolfe F et al (1990) The American College of Rheumatology 1990 criteria for the classification of fibromyalgia. Report of the Multicenter Criteria Committee. Arthritis Rheum 33(2):160–172 10.1002/art.1780330203 2306288

[25] Pollard CA (1984) Preliminary validity study of the pain disability index. Percept Mot Skills 59(3):974 10.2466/pms.1984.59.3.974 6240632

[26] Cohen J (1988) Statistical power analysis for the behavioral sciences, 2nd ed. Routhledge, New York

[27] StataCorp LLC (2017) Stata statistical software: Release 15. StataCorp LLC, College Station, TX

[28] Tlusty GC et al (2026) Symptom burden in cancer survivorship: insights from a survivorship program’s patient-reported outcomes database. Qual Life Res. 10.1007/s11136-026-04321-w PMC13282256 42319622

[29] Glare PA, Costa DJ, Nicholas MK (2022) Psychosocial characteristics of chronic pain in cancer survivors referred to an Australian multidisciplinary pain clinic. Psychooncology 31(11):1895–1903 10.1002/pon.5975 PMC9796565 35661330

[30] Bennedsgaard K et al (2020) Long-term symptoms of polyneuropathy in breast and colorectal cancer patients treated with and without adjuvant chemotherapy. Cancer Med 9(14):5114–5123 10.1002/cam4.3129 PMC7367625 32469145

[31] Nicholas M et al (2019) The IASP classification of chronic pain for ICD-11: chronic primary pain. Pain 160(1):28–37 10.1097/j.pain.0000000000001390 30586068

[32] Hellmann SS et al (2025) Associations of graded chronic non-cancer pain severity with well-being and modifiable lifestyle behaviour: findings from a Danish population-based cross-sectional survey. Eur J Pain 29(10):e70138 10.1002/ejp.70138 PMC12501817 41054870

[33] Sandager MT et al (2024) Health-related quality of life, needs, and concerns among cancer survivors referred to rehabilitation in primary healthcare setting. Acta Oncol 63:76–82 10.2340/1651-226X.2024.19636 PMC11332470 38482717

