# Correction to: Identification of high-impact chronic pain among cancer survivors: a cross-sectional study using the Graded Chronic Pain Scale framework

**DOI:** 10.1007/s00520-026-11300-x

**Published:** 2026-10-06

**Authors:** Julie Roed Jespersen, Henrik Bjarke Vaegter, Caroline Matilde Elnegaard, Lene Jarlbaek

**Affiliations:** 1 Pain Research Group, Pain Center, Department of Anaesthesiology and Intensive Care, Odense University Hospital, Odense, Denmark; 2 Department of Clinical Research, Faculty of Health Sciences, University of Southern Denmark, Odense, Denmark; 3 The Danish Knowledge Centre for Rehabilitation and Palliative Care (REHPA), Odense University Hospital, Nyborg, Denmark; 4 Department of Cardiology, Odense University Hospital, Odense, Denmark

**Correction to: Supportive Care in Cancer (2026) 34:961**

**https://doi.org/10.1007/s00520-026-11190-z**

The published article contains two incorrect effect sizes in the Results section, an incorrect publication year in reference 20, and incorrect abbreviation definitions below Table 1.

In the Results section, “*Comparison of cancer survivors with HICP and matched patients with HICP from the Pain Center*”, the effect size for pain intensity is incorrectly stated as:

“pain intensity (d = 0.27, *p* < 0.01)”

The correct text is:

“pain intensity (d = 1.03, *p* < 0.01)”

In the same paragraph, the effect size for anxiety symptoms is incorrectly stated as:

“GAD-7; d = 0.07, *p* = 0.61”

The correct text is:

“GAD-7; d = 0.05, *p* = 0.61”

In reference 20, the publication year is incorrectly stated as 1976. The correct publication year is 2013. The reference should read:

“Soer, R. et al. (2013). Extensive validation of the pain disability index in 3 groups of patients with musculoskeletal pain. Spine, 38(9), E562–E568. https://doi.org/10.1097/BRS.0b013e31828af21f”

Finally, the abbreviation definitions below Table 1 are incorrectly presented in regards to the PROMIS abbreviation.

Abbreviations: *Q* questionnaire, *REHPA* Danish Knowledge Centre for Rehabilitation and Palliative Care, *NRS* Numeric Rating Scale, *EORTC QLQ-C30* European Organisation for Research and Treatment of Cancer Quality of Life Numeric Rating Scale, *EORTC QLQ-C30* European Organisation for Research and Treatment of Cancer Quality of Life Patient-Reported Outcomes Measurement Information System, *GCPS-R* Graded Chronic Pain Scale–Revised, *HICP* high-impact chronic pain, *ACR* American College of Rheumatology, *PDI* Pain Disability Index, *PCS*Pain Catastrophizing Scale

The correct abbreviation list is:

“Abbreviations: *Q* questionnaire, *REHPA* Danish Knowledge Centre for Rehabilitation and Palliative Care, *NRS* Numeric Rating Scale, *EORTC QLQ-C30* European Organisation for Research and Treatment of Cancer Quality of Life Numeric Rating Scale, *EORTC QLQ-C30* European Organisation for Research and Treatment of Cancer Quality of Life, *PROMIS* Patient-Reported Outcomes Measurement Information System, *GCPS-R* Graded Chronic Pain Scale–Revised, *HICP* high-impact chronic pain, *ACR* American College of Rheumatology, *PDI* Pain Disability Index, *PCS* Pain Catastrophizing Scale”

The original article has been corrected.

