# Supplementary material for: Identification of high-impact chronic pain among cancer survivors: a cross-sectional study using the Graded Chronic Pain Scale framework
Source: Support Care Cancer. 2026 Sep 11;34(10):961. doi: 10.1007/s00520-026-11190-z (PMC13569589; doi:10.1007/s00520-026-11190-z)
Supplement: Supplementary file 1 [file 520_2026_11190_MOESM1_ESM.docx]

**Supplementary contents:**

| **Supplementary Table 1:** ´C-module´responders and non-responders | | | | | | | |
| --- | --- | --- | --- | --- | --- | --- | --- |
|  |  |  | N=270 | | Non responders N=75 | | t-test/Chi^2^, p |
| **Demographics** | **Age (years)** |  |  |  |  |  |  |
|  |  | Mean (95% CI) | 55,2 | (53,9 ; 56,6) | 51,5 | (49 ; 54) | 0,01 |
|  | **Sex** |  |  |  |  |  |  |
|  |  | Female % | 89 |  | 84 |  | 0,25 |
|  | **Marital status** |  |  |  |  |  |  |
|  |  | Single, n % | 84 | 31,1 | 26 | 34,7 | 0,42 |
|  |  | Married/partnered, n % | 185 | 68,5 | 49 | 65,3 |  |
|  |  | Missing, n % | <5 | <5 |  |  |  |
|  | **Educational attainment** | |  |  |  |  |  |
|  |  | Lower secondary or other, n % | 108 | 40 | 30 | 40 | 0,64 |
|  |  | Upper secondary, n % | 125 | 46,3 | 29 | 38,7 |  |
|  |  | Higher education, n % | 33 | 12,2 | 11 | 14,7 |  |
|  |  | Missing, n % | <5 | <5 | 5 | 6,7 |  |
| **Health and QoL** | **Regular smoker** |  |  |  |  |  |  |
|  |  | No, n % | 123 | 45.56 | 34 | 45.33 | 0,63 |
|  |  | No, but previous, n % | 118 | 43.70 | 32 | 42.67 |  |
|  |  | Yes, n % | 29 | 10.74 | 9 | 12 |  |
|  | **Alcohol consumption** | |  |  |  |  |  |
|  |  | 0 units / week, n % | 110 | 40,7 | 35 | 47,3 | 0,38 |
|  |  | 1 - 7 units / week, n % | 124 | 45,9 | 33 | 44,6 |  |
|  |  | 8 + units / week, n % | 36 | 13,3 | 6 | 8.11 |  |
|  | **Physical activity level** | |  |  |  |  |  |
|  |  | Low, n % | 33 | 12,2 | 18 | 24 | 0,03 |
|  |  | Moderate, n % | 191 | 70,7 | 46 | 61,3 |  |
|  |  | High, n % | 46 | 17 | 10 | 13,3 |  |
|  | **Self rated health (PROMIS: 1-6) higher is better** | | |  |  |  |  |
|  |  | Mean (95% CI) | 2,3 | (2,3 ; 2,4) | 2,3 | (2,2 ; 2,5) | 1 |
|  | **Self rated quality of life past week (0-7) lower is worse** | | |  |  |  |  |
|  |  | Mean (95% CI) | 4,1 | (4 ; 4,3) | 4 | (3,7 ; 4,3) | 0,44 |
| **Pain** | **Pain duration** |  |  |  |  |  |  |
|  |  | < 3 months, n% | 23 | 8,5 | <5 | <5 | 0,70 |
|  |  | 3-6 months, n % | 34 | 12,6 | 9 | 12 |  |
|  |  | 6-12 months, n % | 64 | 23,7 | 21 | 28 |  |
|  |  | >1 year, n % | 148 | 54,8 | 41 | 54,7 |  |
|  |  | Missing, n % | <5 | <1 | <5 | <5 |  |
|  | **Pain intensity (last 48 hours)** | |  |  |  |  |  |
|  |  | Mean (95% CI) | 5,5 | (5,3 ; 5,7) | 5,1 | (4,7 ; 5,5) | 0,04 |
|  |  | Missing n, % | <5 | <1 | <5 | <5 |  |
|  |  | Mild (1-3), n % | 25 | 9,3 | 11 | 14,7 |  |
|  |  | Moderate (4-6), n % | 167 | 61,9 | 48 | 64 |  |
|  |  | Severe (>=7), n % | 78 | 28,9 | 16 | 21,3 |  |
|  | **Daily activities limited by pain (past week)** | |  |  |  |  |  |
|  |  | Not at all, n % | 32 | 11,9 | 6 | 8 | 0,63 |
|  |  | A little, n % | 134 | 49,6 | 43 | 57,3 |  |
|  |  | Quite a bit, n % | 82 | 30,4 | 20 | 26,7 |  |
|  |  | Very much, n % | 22 | 8,2 | 6 | 8 |  |

| **Supplementary Table 2:** Demographic and health behaviour data across GCPS impact levels | | | | | | | | | | |
| --- | --- | --- | --- | --- | --- | --- | --- | --- | --- | --- |
|  |  | Total n=270 | | Mild n=34 | | Bothersome n = 70 | | High Impact n=166 | | One-way ANOVA |
| **Age (years)** |  |  |  |  |  |  |  |  |  | p |
|  | Mean (95% CI) | 55,2 | (53,9 ; 56,6) | 56,1 | (52,5 ; 59,7) | 54,7 | (52,1 ; 57,4) | 55,3 | (53,6 ; 57) | 0,83 |
|  | Median (IQR) | 55 | (49, 63) | 56.5 | (48, 63) | 54,5 | (49, 63) | 55 | (49, 63) |  |
| **Sex** |  |  |  |  |  |  |  |  |  |  |
|  | Female% | 88,9 |  | 91,2 |  | 85,7 |  | 89,8 |  | 0,60 |
| **Marital status** |  |  |  |  |  |  |  |  |  |  |
|  | Single, n % | 84 | 31,1 | 7 | 20,6 | 21 | 30 | 56 | 33,7 | 0,23 |
|  | Married/partnered, n % | 185 | 68,5 | 27 | 79,4 | 48 | 68,6 | 110 | 66,3 |  |
|  | Missing, n % | <5 | <5 | 0 | 0 | <5 | <5 | 0 | 0 |  |
| **Educational attainment** | |  |  |  |  |  |  |  |  |  |
|  | Lower secondary or other, n % | 108 | 40 | 14 | 41,2 | 36 | 51,4 | 58 | 34,9 | 0,08 |
|  | Upper secondary, n % | 125 | 46,3 | 15 | 44,1 | 27 | 38,6 | 83 | 50 |  |
|  | Higher education, n % | 33 | 12,2 | 5 | 14,7 | 6 | 8,6 | 22 | 13,3 |  |
|  | Missing, n % | <5 | <5 | 0 | 0 | <5 | <5 | <5 | <5 |  |
| **Regular smoker** |  |  |  |  |  |  |  |  |  |  |
|  | No, n % | 123 | 45,6 | 15 | 44,2 | 36 | 51,4 | 72 | 43,4 | 0,89 |
|  | No, but previous, n % | 118 | 43,7 | 15 | 44,1 | 28 | 40 | 75 | 45,2 |  |
|  | Yes, n % | 29 | 10,7 | <5 | 11,8 | 6 | 8,6 | 19 | 11,4 |  |
| **Alcohol consumption** | |  |  |  |  |  |  |  |  |  |
|  | 0 units / week, n % | 110 | 40,7 | 11 | 32,4 | 29 | 41,4 | 70 | 42,2 | 0,61 |
|  | 1 - 7 units / week, n % | 124 | 45,9 | 18 | 52,9 | 30 | 42,9 | 76 | 45,8 |  |
|  | 8 + units / week, n % | 36 | 13,3 | 5 | 14,7 | 11 | 15,7 | 20 | 12,1 |  |
| **Physical activity level** | |  |  |  |  |  |  |  |  |  |
|  | Low, n % | 33 | 12,2 | <5 | <10 | 10 | 14,3 | 21 | 12,7 | 0,49 |
|  | Moderate, n % | 191 | 70,7 | 26 | 76,5 | 44 | 62,9 | 121 | 72,9 |  |
|  | High, n % | 46 | 17 | 6 | 17,7 | 16 | 22,9 | 24 | 14,5 |  |

**Supplementary Figure 1** Spatial pain distribution. (A) Body chart from the PainData database; (B) body chart divided into 71 body areas; (C) body chart divided into seven body regions [15]
